# Buffalo General Hospital — Surgical Cases Treated

**Published:** 1883-10

**Authors:** Charles C. F. Gay

**Affiliations:** Attending Surgeon


### Clinical Reports.

### BUFFALO GENERAL HOSPITAL—SURGICAL CASES TREATED.

By CHARLES C. F. GAY, M. D., Attending Surgeon.

### SHOT WOUND OF THE RIGHT EYE—EXTIRPATION OF THE GLOBE—BALL FOUND TO HAVE PENETRATED THE LOWER FLOOR OF THE ORBIT AND LODGED IN ANTRUM—RECOVERY.

Mrs. Magee, æt. 25 years, was shot by her husband with a Smith and Wesson pistol, on March 3, 1883, the ball of which —a No. 22 calibre—penetrated the center of the globe of the right eye. She was removed to hospital, and on March 4th, I enucleated the eye and found the ball had taken a downward course, penetrating the lower floor of the orbit, and which was undoubtedly lodged in the antrum. Water dressings were applied and good recovery was made, and as yet no trouble has been experienced from the lodgement of a foreign body in the antrum.

### SHOT WOUND OF THE LEFT EYE—EXTIRPATION OF THE GLOBE OF THE EYE—BALL LODGED ON THE FLOOR OF THE ORBIT, AND REMOVED.

Mr. Magee, æt. 28 years, after shooting his wife, shot himself, the ball penetrating the globe of the eye near its center. The eye was extirpated and the ball, flattened, was found at the near and inner angle of the floor of the orbit, and removed.

Patient made a good recovery.

### TRACHEOTOMY.

Miss Emma Kennedy, æt. 23 years, member of the hospital school for nurses, had been out of the institution a few days nursing two or three children ill with a malignant type of diphtheria. She returned to the hospital feeling ill, complaining of sore throat, etc. Active treatment was at once resorted to. After four days' illness her respiration, of a sudden, became so much obstructed by diphtheritic deposit, that her face became livid, eyes rolled upward, frothing at mouth, pulseless, unconscious, insensible, and presenting the appearance of one gasping for the last time.

Summoned by Dr. Diehl, I reached her bedside in a moment or two, and although the case seemed hopeless and an operation needless, still it was rapidly made, and there being no tracheal tube at hand when the tracheal incision was made, an ordinary silver male catheter was inserted and answered the purpose most admirably. The catheter was held in position until a tracheal tube was obtained; upon inserting the former much mucus, with some membrane, was thrown violently out, and the breathing at once became full, free and easy; the patient's consciousness, color and pulse returned. She remained very comfortable for two days, giving occasional encouragement of ultimate recovery, but at length succumbed to the malignant type of the disease, with consciousness maintained up to within a half hour of her death.

### COMPOUND DEPRESSED FRACTURE—RIGHT PARIETAL AND TEMPORAL BONES—TREPHINING—DEATH IN FORTY-EIGHT HOURS.

Mr. Strausser, æt. 32 years, fell through the hatchway from the second floor of a malt house to the basement, striking on his head.

Entered hospital on March 4, 1883. I saw him within an hour after injury; patient was etherized; a flap laid back revealed the extent of fracture, which was v-shaped, the apex toward the vertex, and depressed. The trephine was employed, a button of bone removed and the depressed fragment of the parietal bone elevated. A piece of the right temporal bone, measuring three-quarters of an inch in diameter, was removed by the rongeur. Patient vomited much blood, and percussion revealed presence of blood in left pleural cavity. Death occurred in forty-eight hours.

### COMPOUND COMMINUTED FRACTURE OF TIBIA.

Wm. Auer, æt. 40 years; German; single; shoemaker; was injured on April 1, 1883. The wheel of a hand-car passed over his right leg. The tibia is fractured near the knee, and also near the ankle, with a small flesh wound. The limb is much swollen, discolored, and looks unpromising for recovery. He entered hospital April 21st. At first, amputation seemed to afford the only surgical resource, but the limb was placed upon a bran pillow in the fracture box, alcohol lotion one-third to two thirds carbolized water employed, and the limb enveloped in oil silk. Improvement was steady under this treatment. May 19th, there is union of bone. The Bavarian splint has been used for the past three days in place of the fracture box. 26th, one side-splint employed only.

June 23d—Patient has been up but cannot remain up. There is delayed union at the upper third of tibia. Applied to-day plaster of Paris splint from foot to above the knee.

This patient recovered with a good limb.

### COMPOUND COMMINUTED FRACTURE OF THE UPPER PORTION OF RIGHT TIBIA—AMPUTATION ABOVE THE KNEE—DEATH.

Dennis Sullivan, æt. 30 years; temperate; single; Ireland; was injured at 12 o'clock, May 18, 1883, by being caught between bumpers on Nickel Plate road, and brought to hospital at 4 P. M. Fracture is high up toward knee; two flesh wounds; there has been much loss of blood, and blood still oozes when compression is removed; pulse feeble.

Patient was etherized, after giving a large dose of brandy, with twenty drops of laudanum. Esmarch's bandage was employed, and assisted by Dr. Putnam, house physician, I amputated the thigh at the lower fourth. Three arteries required ligation. Wound was dressed with boro-glyceride, over which was placed Mackintosh's covering and roller bandage. Operation was done with antiseptic precautions, which were carried out in all their details.

But a few drops of blood were lost at the amputation. Examination of the bones showed that the tibia was broken into thirteen pieces; the fibula was not broken.

19th—Pulse 112; temperature 101°.

20th—Doing well.

On the fifth day after the amputation, I was surprised to learn that the patient was dead; cause supposed to be from exhaustion. I am unable to assign a cause, unless it may be the loss of a large quantity of blood previous to his entry to hospital; but granted that much blood had been lost, still, the fact remains that the patient, on the fourth day after the amputation, was in an apparent good condition, and without any symptom or symptoms that could possibly lead one to suspect a fatal result.

### COMPOUND COMMINUTED FRACTURE OF TIBIA—AMPUTATION OF THIGH—RECOVERY.

Simon Curley, æt. 18 years, trying to board an Erie freight car, fell, and the wheel passed over his left leg, causing a large flesh wound well up toward the knee-joint, splitting and comminuting the tibia. Three small fragments of bone were removed. He was brought to hospital same day of injury at 4 P. M., May 20, 1883. Pulse feeble; face pallid. Stimulants were administered. I proceeded at once to amputate, assisted by Dr. Putnam, house physician. Ether and Esmarch's bandage were employed. Amputation was made at lower fourth of femur. Ligature placed around four arteries; no blood lost during or after operation. Patient almost pulseless. Brandy and ether injected freely hypodermically during the operation.

Antiseptic precautions were employed, with boro-glyceride dressings. Vomited after getting to bed. Both arms were bandaged in order to furnish head and heart with more blood. Hot brandy and laudanum were administered as soon as the stomach could retain them. At 9 A. M. the following morning the patient was in a good condition. Examination of bones showed fibula unfractured, tibia comminuted into five pieces, with long longitudinal fracture extending well up to knee.

May 27th—Wound looks and smells bad. Patient has had specific disease. Ordered iodides of mercury and potassa. Pulse 120; temperature 100¾°. Quinine and laudanum. Iodides used about one week. Wound improved after their use. Wound closed up July 24th, and patient returned to his home soon thereafter.

### COMPOUND COMMINUTED FRACTURE OF TIBIA AND FIBULA—RESECTION OF ENDS OF BOTH BONES—TIBIA WIRED TOGETHER—GOOD RESULT OF CONSERVATIVE SURGERY.

Reported by Dr. Webster, House Surgeon.

James Hallum, æt. 67 years; entered hospital June 5, 1883, with compound comminuted fracture of both bones of the right leg, with extensive flesh wound; says it was caused three days ago by the falling of a large stone upon it while he was engaged in excavating for a railroad. He says six fragments of bone were removed when the wound was first dressed by the local surgeon. Cold water dressings had been applied before the patient entered hospital. Here the wound was thoroughly irrigated and the limb suspended in a tin trough; two days later patient was removed from the ward to a tent on the lawn, the leg placed in fracture box, and dressings of alcohol, one-third part, and carbolic acid one-fortieth, two-thirds applied. Pulse and temperature nearly normal after first day in tent.

June 15th—The ends of both tibia and fibula were resected by Dr. Gay, and ten fragments of bone removed. Ends of tibia drilled and wired together with silver wire; counteropening made upon opposite side of leg and drainage tube passed through; iodoform applied to wound; wound irrigated thoroughly and entire drainage changed each morning; alcohol and carbolic cloths changed as often as they became dry; simple cerate over wound itself; the operation was followed by slight rise in temperature for three or four days, when it again subsided to near the normal, 99–100°.

June 20th—Patient in good spirits; temperature 99° in morning; pulse 88; wound healthy and granulations well marked over nearly the entire surface; new skin closing in from below.

July 13th—Wound closed over bone; silver wire removed a week since; small pieces of bone have exfoliated; put limb in fracture box and laid upon bran bag.

August 20th—Closure of wound and firm body union; shortening of limb two and a half inches; patient able to be up.

### COMPOUND COMMINUTED FRACTURE OF TARSUS—AMPUTATION.

Reported by Dr. Webster, House Surgeon.

C. Leiber, æt 37 years, German; entered hospital June 6, 1883; patient has a crushed right foot, over which has passed a car wheel, laying open the tarsus to the depth of two inches on the external side; circulation in foot and toes good. Boro-glyceride dressing was applied and foot strapped so as to adjust the parts as nearly as possible.

June 9th—Temperature went up to 104½°; foot and leg œdematous; boro-glyceride discontinued and poultices to leg and foot ordered, to be changed or renewed every four hours.

June 10th. Large abscess opened on ankle and three-fourths ounces of pus removed; counter-opening made and drainage tube passed through.

June 12th—Abscess in instep opened and drainage tube introduced; poultice and strapping continued; temperature reduced to 101°, at night after abscesses were opened; leg infiltrated considerably.

June 18th—Leg amputated at middle third by Dr. Gay; drainage tube passed from one angle of wound to the other; flaps united by three silk sutures and adhesive straps between the sutures; patient immediately removed to tent and wound treated antiseptically with boro-glyceride dressings; temperature for the following week 99°, in morning; 100–101°, at night; pulse at night, 90.

June 22d—Drainage tube removed; slight purulent discharge from angle; flaps apparently united; patient sat up three hours this morning, being the fourth day after the amputation.

June 25th—Doing well, no change unless for the better; pulse, 76; temperature, 100¾° at night.

July 13th—Flaps firmly united; patient up and about on crutches; rapid convalescence has been owing, probably, to tent life or out-door air.

